# GABPA-dependent down-regulation of DICER1 in follicular thyroid tumours

**DOI:** 10.1530/ERC-19-0446

**Published:** 2020-03-11

**Authors:** Johan O Paulsson, Na Wang, Jiwei Gao, Adam Stenman, Jan Zedenius, Ninni Mu, Weng-Onn Lui, Catharina Larsson, C Christofer Juhlin

**Affiliations:** 1 Department of Oncology-Pathology, Karolinska Institutet, Karolinska University Hospital, Stockholm, Sweden; 2 Department of Molecular Medicine and Surgery, Karolinska Institutet, Karolinska University Hospital, Stockholm, Sweden; 3 Department of Breast, Endocrine Tumours and Sarcoma, Karolinska University Hospital, Stockholm, Sweden; 4 Department of Pathology and Cytology, Karolinska University Hospital, Stockholm, Sweden

**Keywords:** DICER1, GABPA, follicular, thyroid, carcinoma

## Abstract

Mutations in the miRNA enzyme gene *DICER1* have been reported in several endocrine malignancies and is associated with the rare tumour-predisposing DICER1 syndrome. *DICER1* mutations have been reported in subsets of follicular thyroid carcinoma (FTC), but the role of DICER1 in follicular thyroid tumorigenesis has not been extensively studied. In this study, we investigate the role of DICER1 in 168 follicular thyroid tumours and in an FTC cell line. We found rare *DICER1* mutations in paediatric FTC cases and a general DICER1 down-regulation in FTCs visualized both on mRNA and protein level, especially pronounced in Hürthle cell carcinoma (HuCC). The down-regulation was also evident in follicular thyroid adenomas (FTAs), suggesting a potential early step in tumorigenesis. The expression of *DICER1* was lower in FTCs of older patients in which *TERT* promoter mutations are more frequent. In FTCs, *DICER1* down-regulation was not caused by gene copy number loss but significantly correlated to expression of the transcription factor *GABPA* in clinical cases. GABPA was found to bind to the *DICER1* promoter and regulate *DICER1* expression *in vitro*, as *GABPA* depletion in FTC cell lines reduced *DICER1* expression. This in turn stimulated cell proliferation and affected the miRNA machinery, evident by altered miRNA expression. To conclude, we show that GABPA directly regulates *DICER1* in FTC, acting as a tumour suppressor and displaying down-regulation in clinical samples. We also show reduced expression of *DICER1* in benign and malignant follicular thyroid tumours, suggesting a potentially early tumorigenic role of this gene aberrancy.

## Introduction

Thyroid cancer is the most common endocrine malignancy and accounts for about 1–3% of all new cases of cancers and occurs predominantly in women (Siegel* et al.* 2018). The incidence of thyroid cancer has steadily increased for the last few decades, especially since the beginning of the 1990s, but the mortality rate has remained relatively stable (Kitahara & Sosa 2016). The increase is mainly attributable to the increasing incidence of papillary thyroid carcinoma (PTC), but all forms show an increase (Davies & Welch 2014, Carlberg* et al.* 2016, Kitahara & Sosa 2016, Cronin* et al.* 2018). Follicular thyroid carcinoma (FTC) is a well-differentiated malignancy that accounts for about 10–15% of all thyroid cancers (Aschebrook-Kilfoy* et al.* 2011). It shows a 5-year survival of about 90% but a 10-year survival around 80% (James* et al.* 2016). FTCs are subclassified as minimally invasive (miFTC), encapsulated angioinvasive (eaiFTC) and widely invasive (wiFTC) depending on the histopathologic features such as capsular and/or vascular invasion as specified by World Health Organization (WHO) guidelines from 2017 (Lloyd *et al*. 2017). FTCs with an oxyphilic phenotype are classified as Hürthle cell carcinomas (HuCC), and these show a worse clinical outcome (Lloyd *et al*. 2017).

There are several well-established genetic driver events behind the development of follicular thyroid tumors. Somatic mutations in the *RAS* family are commonly found in both follicular thyroid adenomas (FTAs) and FTCs (*HRAS*, *KRAS* and* NRAS*) (Lemoine* et al.* 1989, Suarez* et al.* 1990, Esapa* et al.* 1999), showing a frequency of approximately 60% in FTC (Fukahori* et al.* 2012). The *PAX8/PPARG* gene fusion is another well-established genetic event found in 30–60% of FTCs (Kroll* et al.* 2000, Marques* et al.* 2002, Nikiforova* et al.* 2003). Activation of the PI3K/Akt pathway have also been observed in FTC (Hou* et al.* 2007), and recent studies have also identified mutations and aberrant methylation in the telomerase reverse transcriptase (*TERT*) promoter region, as well as *TERT* copy number gains and expression which have also been associated with a poor prognosis (Liu* et al.* 2014, Wang* et al.* 2014, Liu & Xing 2016, Paulsson* et al.* 2018).

The mechanisms of tumorigenesis in a substantial subset of FTCs are still unknown, therefore recent studies have utilized next-generation sequencing in an attempt to identify additional genetic changes in FTC. In addition to known driver genes, *DICER1* was found recurrently mutated in 7–10% of FTCs (Yoo* et al.* 2016, Nicolson* et al.* 2018). The *DICER1* gene, located on chromosome 14q32.13, is comprised of 27 exons (ENST00000343455.7) and encodes a highly conserved protein (DICER1 in *Homo sapiens*) that functions as an RNase III enzyme involved in the maturation of miRNA (Bernstein* et al.* 2001, Foulkes* et al.* 2014). In recent years there have been reports of *DICER1* gene mutations in several endocrine malignancies, including thyroid cancer (Solarski* et al.* 2018). The somatic *DICER1* gene mutations reported in PTC have been predicted to have a high impact on the function of the DICER1 protein (Cancer Genome Atlas Research Network 2014, Costa* et al.* 2015). Most of the reported somatic mutations are concentrated to the RNase IIIb region (exon 24 and 25), which is in contrast to the germline *DICER1* gene mutations that are found spread out across the gene, resulting in a phenotype predisposed for tumours such as pleuropulmonary blastoma, Sertoli-Leydig cell tumour, multinodular goitre and thyroid cancer, collectively called DICER1 syndrome (Hill* et al.* 2009, Slade* et al.* 2011, Foulkes* et al.* 2014). These findings suggest that DICER1 alterations is an important factor for the development of both benign and malignant thyroid nodules. Moreover, a recent study of PTC clinical samples and cell lines proposed that the transcription factor GA binding protein (GABPA) regulates *DICER1* transcriptionally (Yuan *et al*. 2019). We therefore aimed to establish the contribution of *DICER1* dysregulation in follicular thyroid tumorigenesis and to examine the relationship between *DICER1* and GABPA in a large clinical material as well as in established FTC cell lines.

## Materials and methods

### Patient information and tumour specimen

Informed consent was collected before the study was carried out and the study was approved by the Ethical Review Board (Etikprövningsmyndigheten, Stockholm). The samples were collected from the Karolinska University Hospital biobank and included patients operated at Karolinska University Hospital between the years 1986 to 2017. All cases were diagnosed by an endocrine pathologist and later re-classified by an additional independent endocrine pathologist according to the WHO 2017 guidelines (Lloyd *et al*. 2017). In total, the cohort included 168 patients diagnosed with either follicular thyroid adenoma (FTA) (*n* = 43), follicular tumour of uncertain malignant potential (FT-UMP) (*n* = 7), HuCC (*n* = 24) or FTC (*n* = 94). Non-neoplastic thyroid tissues used for endogenous control experiments included ten cases of normal thyroid tissue obtained from patients with benign thyroid or parathyroid disease and six cases of multinodular goitre. Normal thyroid tissue was also present adjacent to FTCs/HuCCs in subsets of the slides used for immunohistochemical analyses and used as internal references.

### Cell lines and culture conditions

Human follicular thyroid carcinoma cell lines FTC-133 (derived from a lymph node metastasis) and FTC-238 (derived from a lung metastasis) (product numbers 94060901 and 94060902 respectively, Sigma) were included in this study. The mutational landscape of both cell lines was recently published (Landa* et al.* 2019), and we confirmed the presence of a homozygous *TERT* promoter mutation c.228C>T (-124C>T) by Sanger sequencing in both cell lines (data not shown). The cell lines are identical to the source provided by the depositor based on an STR-PCR analysis. Cells were maintained in DMEM supplemented with 5% foetal bovine serum (FBS) in culture dishes. Experiments were conducted after 12–15 passages, allowing a maximum of 15 passages.

### siRNA treatment, *DICER1* and *GABPA* overexpression

For *GABPA* and* DICER1* silencing, 1 × 10^5^ cells were transfected with 25 pmol siRNA. The GABPA siRNAs are entitled siGABPA#1 and siGABPA#2; both customized as described previously from Thermo Fisher Scientific (Yuan* et al.* 2019). For *DICER1*, the siRNAs were entitled siDICER1#1 and siDICER1#2 (siDICER1#1 customized as previously described from Invitrogen (Yuan* et al.* 2019) and commercially available siDICER1#2, product number #6576, Cell Signaling Technology). In addition, a control siRNA (Sigma) was used. Cells were transfected with Lipofectamine 3000 (Thermo Fisher Scientific) according to the protocol provided by the manufacturer. Sequences for siRNAs are listed in Supplementary Table 1 (see section on supplementary materials given at the end of this article).

Plasmid (pCMV6entryHisDICER) for *DICER1* overexpression was constructed based on pDESTmycDICER (Addgene, Cat#19873). Restriction enzymes Not I, Sal I, and HindIII were used to digest pDESTmycDICER, and target backbone, pCMV6entry was treated with Not I and Sal I. Purified DICER1 ORF, as well as pCMV6entry, after electrophoresis were ligated by T4 DNA ligase. Sanger sequencing confirmed the pCMV6entryHisDICER construction. All enzymes were purchased from New England Biolabs, and *E. coli* DH5α were used to amplify the plasmid. For *DICER1* overexpression, 8 × 10^4^ cells were grown with 70–80% confluence on 6-well plates and then transfected with either 2 µg/well pCMV6-entry DICER expression plasmid or empty vector using Lipofectamine 3000 (Thermo Fisher Scientific) according to the protocol provided by the manufacturer. The construct was validated by Sanger sequencing at the KIGene core facility.

For the *GABPA* overexpression, the plasmid construct has been previously described in detail, and the methodology followed the same principles as for *DICER1*, except for the usage of Lipofectamine LTX instead of Lipofectamine 3000 as a transfection agent (Yuan* et al.* 2019).

### Sanger sequencing

In total, 61 FTCs, 15 HuCCs, 43 FTAs and 7 FT-UMPs were analysed for the *DICER1* hot spot mutations in codon 1705, 1709, 1810, 1813 and 1814 using Sanger sequencing with primers specified in Supplementary Table 1. The tumours were also analysed for the mutations reported in the whole-exome sequencing study (Nicolson* et al.* 2018), codons 290 and 304, with primers specified in Supplementary Table 1. The chromatograms were aligned to reference sequence and manually analysed using Genome Compiler (Twist Bioscience, San Francisco, CA, USA) and CodonCode Aligner (CodonCode Corporation, Centerville, MA, USA).

### Copy number alterations

Copy number (CN) alterations of the *DICER1* gene locus was assessed in 48 FTCs and 12 HuCCs. The samples were analysed using the TaqMan assays (Applied Biosystems), Hs02002254_cn for *DICER1* and TaqMan Copy number reference assay *RNaseP* as endogenous control. The CN was calculated using CopyCaller v.2.1 (Applied Biosystems). Six cases of multinodular goitre were included as calibrator samples in every run, and additionally, three normal thyroid samples were also used as diploid controls. CN gain was defined as three or more copies and CN loss as one copy or less.

### mRNA expression analyses

Total RNA from human thyroid tissue and cell lines was extracted with mirVana RNA Isolation kit (Applied Biosystems) and cDNA synthesized using High-Capacity cDNA Reverse Transcription Kit (Applied Biosystems). Quantitative real-time PCR (qRT-PCR) was performed in FTC-238 cells, 42 FTAs, 49 FTCs and 13 HuCCs. Ten non-cancerous thyroid tissue samples were used as references. *DICER1* and *GABPA* mRNA expression levels and endogenous control β-actin were investigated using SYBR Green with ABI 7900HT real-time PCR system (Applied Biosystems). Samples were run in triplicates, and the relative expression levels of *GABPA* and *DICER1* were quantified by normalization to the housekeeping gene β-actin and calculations based on 2^−ΔCt^ in tissues and 2^−ΔΔCt^ in cell lines. Primer sequences are listed in Supplementary Table 1.

### miRNA expression

Total RNA was extracted from cell lines using the mirVana RNA Isolation kit (Applied Biosystems) and cDNA was synthesized using MicroRNA cDNA Synthesis kit (Applied Biosystems) with assay specific TaqMan primers, *miR-34a-5p* (hsa-miR-34a, 000425), *miR-199a* (hsa-miR-199a, 000498) and the control assay RNU6B (001093) (Applied Biosystems). Samples were run in StepOnePlus Real-Time PCR System (Applied Biosystems) in triplicates with assay specific TaqMan primers stated previously and expression levels were calculated using the 2^−ΔΔCt^.

### Immunohistochemistry

In total, 44 cases of FTCs and seven cases of HuCCs were subject to immunohistochemical staining. The tissue slides included corresponding normal thyroid tissue for 40 cases. All cases were formalin-fixed paraffin-embedded, cut into 4 μm thick sections and stained using the Envision+ Dual Link System-HRP (DAB+) (DAKO). The tissue slides were incubated with the Anti-Dicer MAB (ab14601, Abcam, diluted 1:100) for 30 min at room temperature before proceeding with the DAKO staining according to the manufacturer’s instructions. The slides were counter-stained with haematoxylin for 5 min and then mounted. The stained slides were subsequently evaluated by an experienced endocrine pathologist and scored as either ‘negative’ (completely devoid of immunoreactivity), ‘weak/focal’ (weak staining in subsets of cells), ‘intermediate’ (diffuse staining of moderate intensity) or ‘strong’ (diffuse staining of strong intensity).

### Immunoblot

Protein pellets were extracted with NP40 lysis buffer and 30 µg of protein was used. The membranes were incubated with the following primary antibodies: Anti-NRF2A/GABPA (rabbit polyclonal antibody ABE1047, Millipore, at 1:1000 dilution) and anti-DICER (rabbit MAB D38E7, Cat# 5362, Cell Signaling Technology, at 1:1000 dilution in 5% non-fat milk). Anti-β-actin (mouse MAB from Sigma, clone A1978) or GAPDH (rabbit MAB, clone 14C10, Cell Signaling Technology) signal served as loading controls.

### Chromatin immunoprecipitation

Chromatin immunoprecipitation (ChIP) assay was performed using SimpleChIP kit (#9005, Cell Signaling Technology). FTC-238 cells were cross-linked in 1% formaldehyde-containing medium and then sonicated to make soluble chromatin. Anti-GABPA and anti-IgG were used to precipitate DNA fragments bound by GABPA. The reverse-cross-linked DNA was used for PCR amplification and subsequent sequencing. The GABPA predicted binding sites of the *DICER1* promoter have been previously published (Yuan* et al.* 2019). The PCR primers for the *DICER1* promoter are listed in Supplementary Table 1, and a detailed ChIP protocol is available on request.

### Cell proliferation analyses

Approximately 8 × 10^4^ FTC-238 cells were seeded into 6-well plates, treated with *DICER1* depletion and *DICER1* overexpression, respectively, stained with Incucyte Nuclight Rapid Red Reagent for cell labelling (Sartorius, Göttingen, Germany) and placed in an incubation chamber maintained at 37°C. Real-time proliferation of FTC-238 cells was photographed and analysed by the IncuCyte S3 Live Cell Imaging and Analysis System (Essen Bioscience, Ann Arbor, MI, USA) through quantitatively detecting alive cells up to 72 h from start. The changes in living cell number represent the cell proliferation.

### Trypan blue exclusion assay

Approximately 8 × 10^4^ FTC-238 cells were seeded into 6-well plates, treated with control and *DICER1* depletion via siRNA, respectively, then cells were collected at 24 h, 48 h and 72 h, stained with trypan blue and counted with a TC10 Automated Cell Counter (BioRad) for cell growth.

### Analysis of apoptosis by fluorescence-activated cell sorting (FACS)

*GABPA*-depleted FTC-238 cells and controls were analysed by fluorescence-activated cell sorting (FACS). Treated and untreated FTC-238 cells were stained with Annexin-V conjugated to fluorescein isothiocyanate (FITC) and propidium iodide (PI) (BioLegend, San Diego, CA, USA) according to the manufacturer’s instructions. Briefly, cells were suspended in 400 µL of Annexin V binding buffer and incubated with 5 µL of FITC Annexin-V and 10 µL of PI for 15 min at room temperature in darkness. After staining, the cells were analysed in a flow cytometer using 488-nm excitation and a 525-nm band pass filter for FITC and a 620-nm filter for PI detection. The percentage of cells undergoing apoptosis were determined by dual-colour analysis. Results were obtained from three independent experiments.

### Statistical analyses

Categorical and binary variables were presented as number (*n*) and proportion (%) of cases, respectively. Chi-square and Fisher’s exact tests were used to compare categorical variable differences. Kaplan–Meier was used to plot overall survival (the percentage of patients in our study who are still alive for a certain period of time after they were diagnosed with FTC/HuCC), disease-free survival (the length of time after primary treatment for FTC/HuCC without known recurrence) and/or disease-specific survival (the percentage of people in our study who have not died from FTC/HuCC in a defined period of time) in cancer patients with low and high *DICER1* mRNA expression (first and fourth quartile, respectively) and log-rank test was used to calculate statistical significance. The differences in *DICER1* and *GABPA* mRNA levels between tumours and normal thyroid tissues were analysed using the Mann–Whitney *U* test. Comparison of expression in *DICER1* and *GABPA* mRNA between control and treated group in FTC cell lines was analysed using Student’s *t*-test. Comparison of cell proliferation between control and treated group in FTC cell lines was analysed using Student’s *t*-test and two-way ANOVA analysis. The Pearson and Spearman correlation tests were used to calculate the correlation between continuous variables. Calculations and graphs were prepared in SPSS version 25 (IBM SPSS Statistics, IBM) and GraphPad Prism version 8 (GraphPad Software). *P* values ≤0.05 were considered statistically significant.

## Results

### Patient characteristics

The clinical characteristics of the patients included in the study are summarized in Table 1. A total number of 168 patients were included in the study. The mean age at surgery was 51, 49, 54 and 59 years for FTA, FT-UMP, FTC and HuCC, respectively. The FTA and FT-UMP groups included 15 and four Hürthle cell tumours, respectively, whereas the HuCCs were separated from the FTC group. There was no relapse in the FTA group but one relapse in the FT-UMP group. The FTC group included eight patients with metastatic disease, and 11 patients that died from the disease but none with metastatic disease. The HuCC group included seven patients who died from the disease but no additional patients with metastatic disease.

**Table 1:** Clinical characteristics of included cases.

Parameter	FTA (*n* = 43)		FT-UMP (*n* = 7)		FTC (*n* = 94)		HuCC (*n* = 24)	
	Observation	No of inf. (*n*)	Observation	No of inf. (*n*)	Observation	No of inf. (*n*)	Observation	No of inf. (*n*)
Mean age at diagnosis, years (min–max)	51 (25–87)	43	49 (30–75)	7	54 (10–91)	94	59 (29–80)	24
Female patients, *n* (%)	31 (72.1)	43	7 (100)	7	60 (63.8)	94	16 (66.7)	24
Mean tumor diameter, mm (min–max)	32 (14–60)	43	33 (15–70)	7	43 (15–100)	90	43 (20–80)	24
WHO 2017 subtype						93		24
Minimally invasive (miFTC), *n* (%)	–		–		40 (43.0)		9 (37.5)^a^	
Encapsulated angioinvasive (eai FTC), *n* (%)	–		–		13 (14.0)		5 (20.8)^a^	
Widely invasive (wiFTC), *n* (%)	–		–		40 (43.0)		10 (41.7)^a^	
T category						91		24
pT1	–		–		11 (12.1)		2 (8.3)	
pT2	–		–		28 (30.8)		8 (33.3)	
pT3	–		–		52 (57.1)		13 (54.2)	
pT4	–		–		0 (0)		1 (4.2)	
Hürthle cell, *n* (%)	15 (34.9)	43	4 (57.1)	7	0 (0)	94	24 (100)	24
Extrathyroidal growth, *n* (%)	–		–		7 (7.6)	92	4 (16.7)	24
Mean Follow-up time, months (min-max)	70 (23–140)	43	104 (9–336)		98 (7–389)	94	95 (13–328)	24
Outcome		43		7		94		24
AWOD, *n* (%)	40 (93)		5 (71.4)		64 (68.1)		15 (62.5)	
AWD, *n* (%)	0 (0)		1 (14.3)		8 (8.5)		0 (0.0)	
DOD, *n* (%)	0 (0)		0 (0)		11 (11.7)		7 (29.2)	
DOOC, *n* (%)	3 (7)		1 (14.3)		11 (11.7)		2 (8.3)	

^a^Subtypes as defined for FTCs. No such subtyping is recommended for HuCCs according the 2017 WHO but are listed here for clarity.

AWD, alive with disease; AWOD, alive without disease; DOD, dead of disease; DOOC, dead of other cause; FTA, follicular thyroid adenoma; FTC, follicular thyroid carcinoma; FT-UMP, follicular tumour of uncertain malignant potential; HuCC, Hürthle cell carcinoma; No of inf., Number of informative cases.

### *DICER1* mutations in follicular thyroid tumours

To determine the occurrence of *DICER1* mutations in follicular thyroid tumours, a total number of 126 tumours were sequenced for mutations in regions previously reported in thyroid cancer. The results are illustrated in Supplementary Fig. 1. Across the cohort, three mutations in two cases were observed. The first case showed a missense mutation at position c.5428G>T (p.D1810Y) and, in addition, a nonsense mutation at position c.911C>A (p.S304X). The patient was a 14-year-old girl diagnosed with miFTC, and these mutations have been previously published for this patient and verified as somatic (Nicolson* et al.* 2018). She exhibited no family history of the DICER1 syndrome. The second patient with a *DICER1* mutation showed a missense mutation at position c.5437G>C (p.E1813Q). This patient was a 10-year-old girl diagnosed with a wiFTC. No constitutional DNA was available for testing whether this variant was constitutional or somatic in origin; however, no family history indicative of the DICER1 syndrome was evident.

### *DICER1* copy number alterations in FTC/HuCC

The gene copy number of the *DICER1* locus was determined in 48 FTCs and 12 HuCCs, six multinodular goitre samples and three normal thyroids. Two of the 48 FTCs displayed three copies of the *DICER1* gene locus (CN gain) and one HuCC showed one copy (CN loss) (data not shown). No copy number aberrations were found in the non-neoplastic group (*n* = 9) of normal thyroid and multinodular goitre samples. The HuCC case with CN loss displayed low levels of *DICER1* mRNA expression.

### Down-regulation of *DICER1* mRNA and protein expression

**Figure 1:**
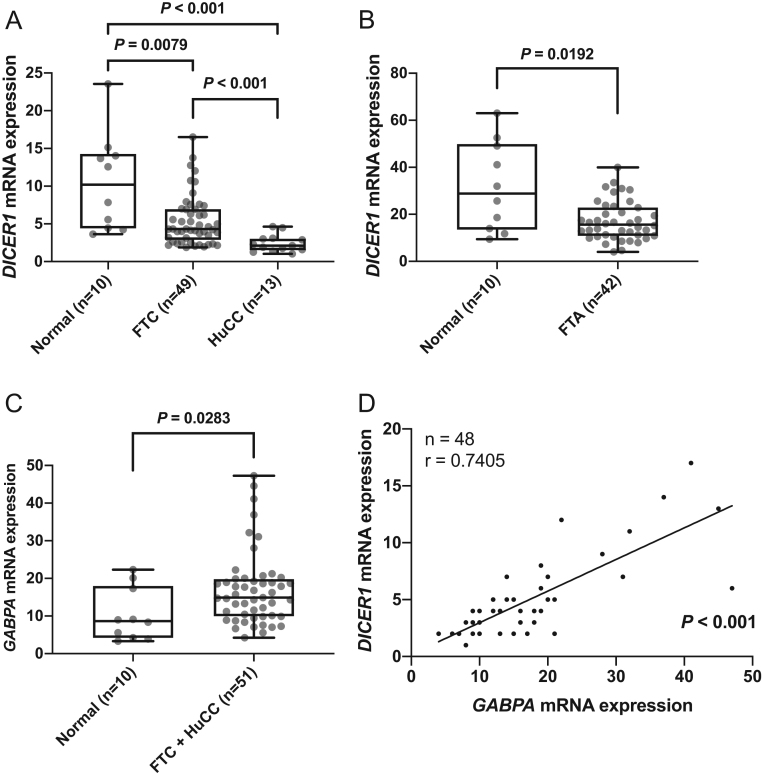
Relative *DICER1* and *GABPA* mRNA expression and their correlation in follicular thyroid carcinomas (FTC), Hürthle cell carcinomas (HuCC) and follicular thyroid adenomas (FTA). (A) *DICER1* mRNA expression in normal thyroid tissue vs FTC and HuCC. (B) *DICER1* mRNA expression in normal thyroid samples vs FTAs. (C) *GABPA* mRNA expression in normal thyroid tissue vs FTC/HuCC. (D) Correlation of* DICER1* mRNA expression and *GABPA* mRNA expression in FTC/HuCC. *P* values were calculated with Mann–Whitney *U* test, Spearman’s and Pearson correlation. Significant *P* values are in bold.

**Figure 2:**
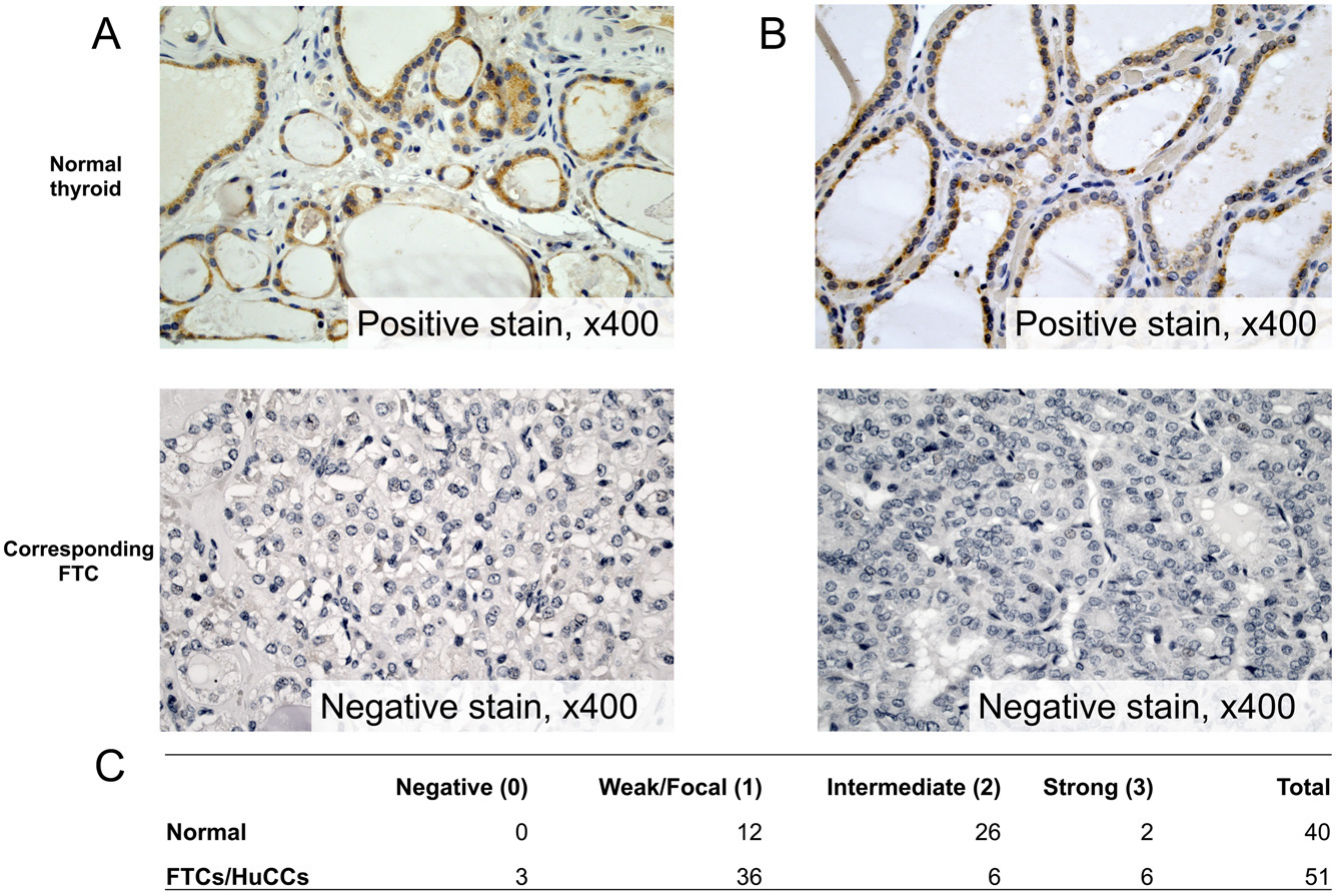
Representative images of the DICER1 immunohistochemistry staining and summary of the staining results. (A and B) The staining of DICER1 in two cases of FTC shows positive staining of the corresponding normal tissue and negative staining in the central parts of the tumour. The tumour tissue, in general, showed weaker DICER1 immunoreactivity compared to the corresponding normal thyroid tissue. Magnification ×400. (C) Summarized results from the immunohistochemical scoring of normal thyroid tissue, follicular thyroid carcinomas (FTC) and Hürthle cell carcinomas (HuCC). A full colour version of this figure is available at https://doi.org/10.1530/ERC-19-0446.

The mRNA expression of* DICER1* was determined in 49 FTCs, 13 HuCCs, 42 FTAs and ten normal thyroid tissues. FTCs and HuCCs displayed lower levels of *DICER1* mRNA compared to normal thyroid tissue (Fig. 1A, *P* = 0.0079 and *P* < 0.001 respectively). *DICER1* mRNA expression levels were lower in HuCCs as compared to FTCs (Fig. 1A, *P* < 0.001). The *DICER1* mRNA expression levels were significantly lower also in FTA compared to normal tissue (Fig. 1B, *P* = 0.0192). To visualize the DICER1 expression pattern, a total of 51 cancerous samples (44 FTC, seven HuCC) and 40 matched normal thyroid samples were stained for DICER1 and graded in a semi-quantitative manner. In a majority of cases, a cytoplasmic staining was observed. Lower DICER1 immunoreactivity in cancerous tissue compared to normal thyroid tissue was observed (*P* < 0.001), as calculated with Mann–Whitney *U* test in a semi-quantitative method. Figure 2 shows two representative cases, where the expression in the central parts of the tumours is lost and the corresponding normal thyroid tissue shows positive cytoplasmic staining.

### *GABPA* mRNA expression is correlated to *DICER1* mRNA expression in FTC/HuCC

Given earlier observations of *DICER1* and *GABPA* correlation in PTC, we explored the relationship between these in FTC/HuCC. *GABPA* mRNA expression was slightly lower in normal thyroid tissue compared to FTC/HuCC (Fig. 1C, *P* = 0.0283). Based on expression data in 39 FTCs and nine HuCCs, the *DICER1* and *GABPA* mRNA expression showed a positive correlation (Fig. 1D).

### *DICER1* expression is reduced after *GABPA* silencing in FTC-238

**Figure 3:**
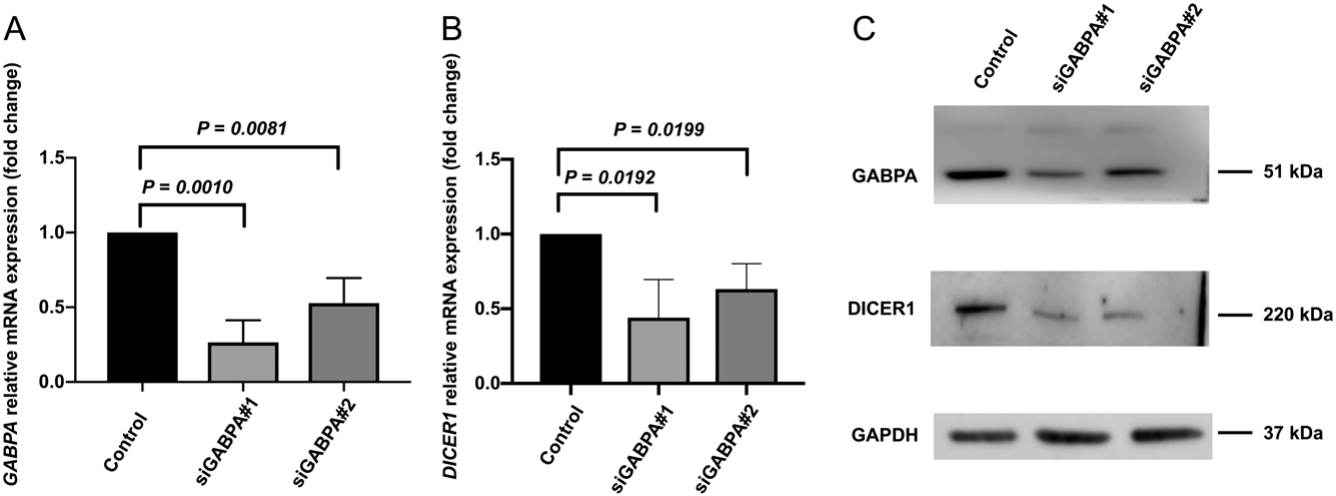
*GABPA* knock down in FTC-238 cell line. (A) *GABPA* mRNA expression in the knock down cells (siGABPA#1 and siGABPA#2) is significantly reduced compared to control cells. (B) *DICER1* mRNA expression in the knock down cells (siGABPA#1 and siGABPA#2) is significantly reduced compared to control cells. (C) Confirmation of reduced levels of *GABPA* and *DICER1* after *GABPA* knock down. Estimated protein sizes are indicated to the right in kilodaltons (kDa). *P* values were calculated with Student’s *t*-test. Significant *P* values are in bold.

As our clinical samples showed a positive correlation between *GABPA* mRNA expression and *DICER1* mRNA expression in thyroid cancer tissues, we sought to explore the relationship between these in FTC cell lines. *GABPA* was knocked down using specific siRNAs and subsequent *DICER1* expression was assessed in FTC cell lines. The efficiency of inhibition of *GABPA* expression in siRNA treated cells was verified using qRT-PCR and Western blot. Successful inhibition was achieved in FTC-238 and subsequent experiments were performed in this cell line. As shown in Fig. 3A, B and C, *GABPA* and *DICER1* mRNA expression and protein expression decreased significantly in *GABPA* depleted FTC-238 cells.

### GABPA overexpression induces augmented DICER1 protein expression

By transfecting FTC-238 cells with a GABPA expression plasmid, increased DICER1 protein expression was evident by Western blot analyses (Supplementary Fig. 2). The results were conducted using two different plasmid concentrations, and visible up-regulation of DICER1 was evident in both experiments.

### GABPA interacts with *DICER1* through binding the *DICER1* promoter

**Figure 4:**
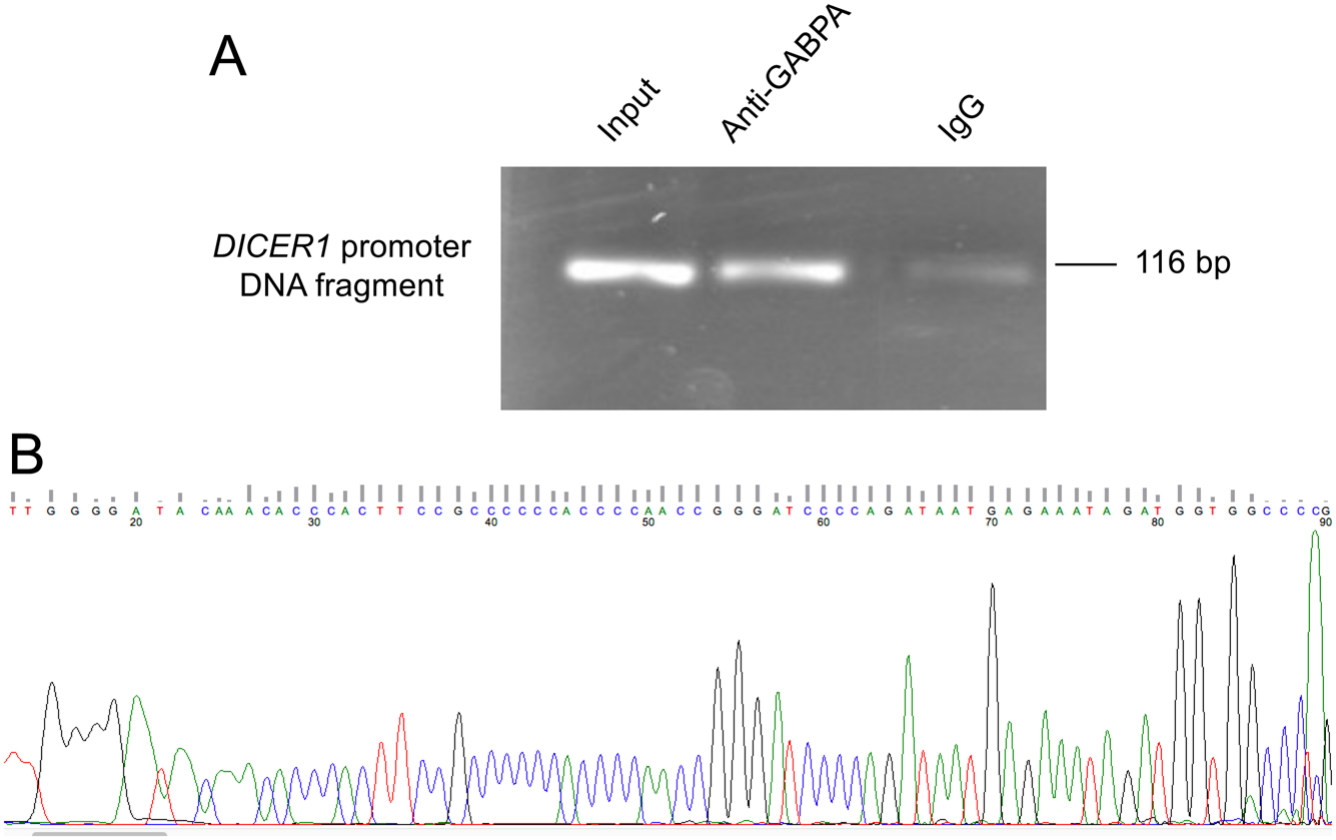
Chromatin immunoprecipitation assay of the binding of GABPA to the *DICER1* promoter. (A) Gel electrophoresis showing DNA bands from *DICER1* promoter PCR product. Lane 1 shows band from 2% input DNA, lane 2 shows band from pulled down DNA using GABPA antibody and lane 3 shows PCR product from cross-linked chromatin DNA incubated with normal rabbit IgG. (B) Chromatogram from the GABPA antibody pulled down DNA and subsequent amplification and sequencing of the *DICER1* promoter using the reverse primer as sequencing primer. The sequence was blasted with a 100% match in the *DICER1* promoter. A full colour version of this figure is available at https://doi.org/10.1530/ERC-19-0446.

To further explore the interaction between GABPA and *DICER1,* a ChIP was performed in FTC-238 cells. After pulling down the DNA-protein complex with anti-GABPA antibody, a selected area of the *DICER1* promoter was amplified using primers specific to the *DICER1* promoter region and sequenced (Fig. 4A and B), showing a physical interaction between the transcription factor GABPA and *DICER1*.

### *DICER1* silencing and overexpression in the FTC-238 cell line alters the proliferation rate

**Figure 5:**
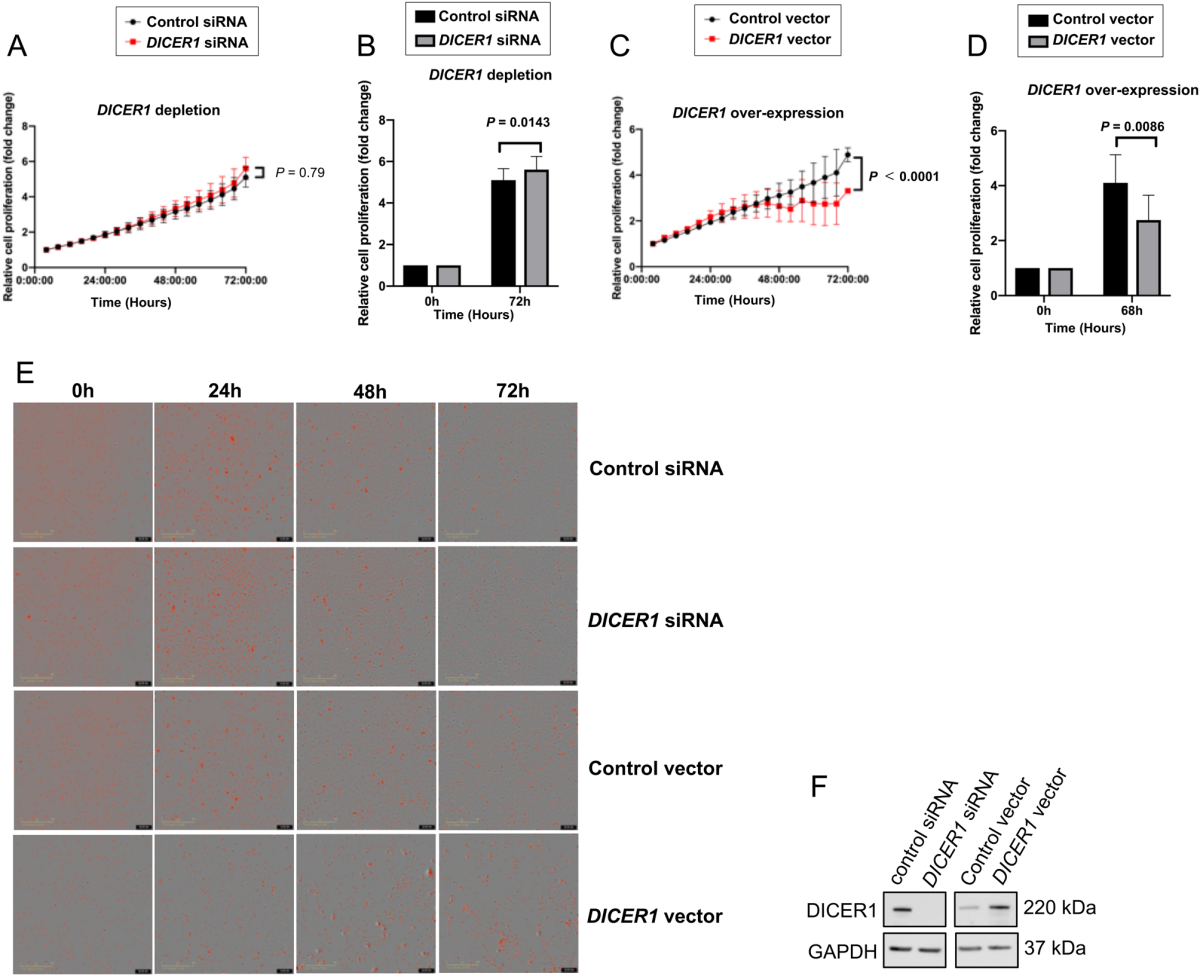
FTC-238 cell proliferation was assessed by monitoring alive cells in real time (Incucyte). Cell proliferation increased after DICER1 depletion using siDICER1#1 (A and B) and decreased in the *DICER1* overexpression experiments (C and D). Difference in time and effect factors was analysed by two-way ANOVA. (B and D) Shows the relative increase and decrease in cell proliferation of FTC-238 cells at the starting and end points, respectively. Cell proliferation was calculated based on the cell number counting from three independent cell experiments, and relative cell proliferation was presented by comparing to cell numbers at the starting point. Date shown are the mean ± s.d. (*n* = 3). Images in E represent the FTC-238 cells growth pattern at different time points, where the red signal indicates the stained cell nuclei. As shown in F, *DICER1* depletion and overexpression experiments were confirmed by Western blot. Estimated protein sizes are indicated to the right. A full colour version of this figure is available at https://doi.org/10.1530/ERC-19-0446.

To explore the proliferative effects of *DICER1,* FTC-238 cells were treated with *DICER1* siRNA and an *DICER1* overexpression vector, respectively. Cell proliferation was assessed at different time points in real time (Fig. 5A, B, C, D and E). Increased cell proliferation was observed in *DICER1* siRNA treated cells compared to the control (Fig. 5B), and likewise, decreased proliferation was evident in *DICER1* overexpression experiments (Fig. 5C and D). The real-time growth pattern of FTC-238 cells at different time points was also visualized (Fig. 5E). *DICER1* knock-down and overexpression was confirmed with Western blot (Fig. 5F).

### Cell viability is increased upon *DICER1* depletion visualized by Trypan blue assay

Analyses of *DICER1* depleted FTC-238 cells collected at 24 h, 48 h and 72 h revealed an increase in viable FTC-238 cells at all three time points observed (Supplementary Fig. 3).

### Analysis of apoptosis by fluorescence-activated cell sorting (FACS)

To study an eventual *GABPA* mediated effect on apoptosis in FTC-238 cells, *GABPA* depleted cells were analysed using fluorescence-activated cell sorting (FACS). As illustrated in Supplementary Fig. 4, there were no evident differences in the number of cells in early or late stages of apoptosis between control cells and *GABPA* knocked-down cells.

### *miR-34a-5p* is dysregulated after silencing of *GABPA* and *DICER1*

**Figure 6:**
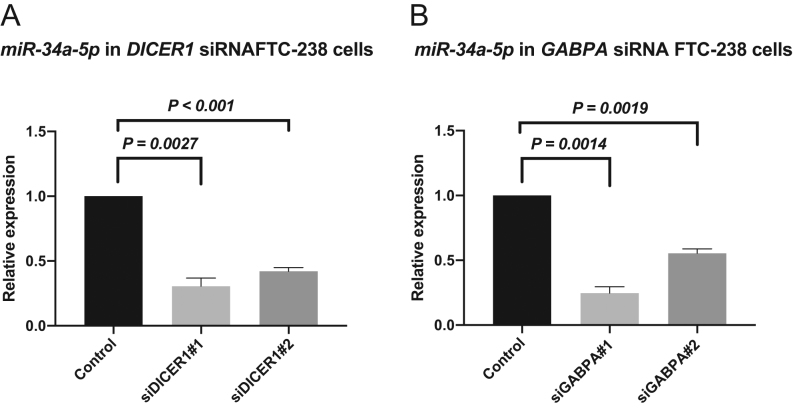
miRNA expression after *GABPA* and *DICER1* knock-down cells respectively. (A) *miR-34a-5p* expression in *DICER1* knock-down FTC-238 cells is reduced (B) *miR-34a-5p* expression in GABPA knock-down FTC-238 cells is also reduced.

To evaluate the consequences of DICER1 down-regulation on the miRNA machinery, the expression for two selected miRNAs was assessed based on previous reports of dysregulation (Rossing* et al.* 2012, Mancikova* et al.* 2015). The *miR-199a-5p* repeatedly did not show any expression in control or siRNA treated cells (data not shown). In contrast, *miR-34a-5p* was down-regulated in both *DICER1* knock-down cells (Fig. 6A) and *GABPA* knock-down cells (Fig. 6B). We also employed the mIRDB database (http://mirdb.org) to mine for predictive targets of *miR-34a-5p*. A total of 899 potential targets were listed, of which we list the top 20 candidates in Supplementary Table 2.

### Clinical outcome in low *DICER1* expressing cases

**Figure 7:**
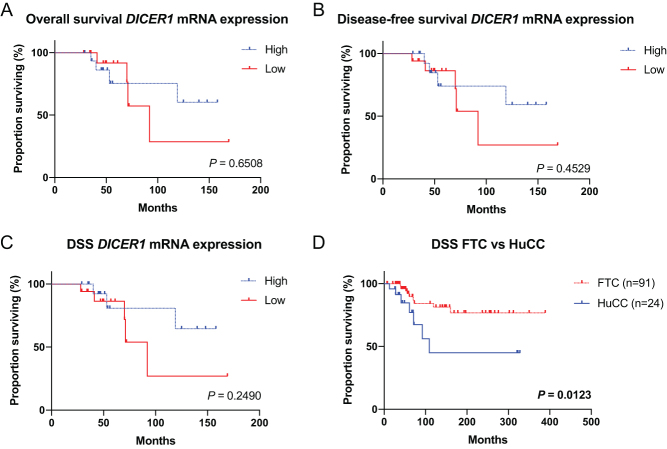
Kaplan–Meier survival curves in follicular thyroid carcinoma (FTC) and Hürthle cell carcinoma (HuCC) patients. (A) The overall survival in low vs high *DICER1* expression. (B) The disease-free survival in low vs high *DICER1* expression. (C) The disease-specific survival (DSS) in low vs high *DICER1* expression. (D) DSS in FTC vs HuCC. *P* values were calculated using the Log-rank test. Significant *P* values are in bold. A full colour version of this figure is available at https://doi.org/10.1530/ERC-19-0446.

Overall survival, disease-free survival and disease-specific survival were not significantly lower for low *DICER1* expressing patients (Fig. 7A, B and C). However, as observed in Fig. 1A the *DICER1* mRNA expression was lower in HuCC, as was the disease-specific survival (Fig. 7D, *P* = 0.0123).

### Age-dependent associations to *DICER1* expression

**Figure 8:**
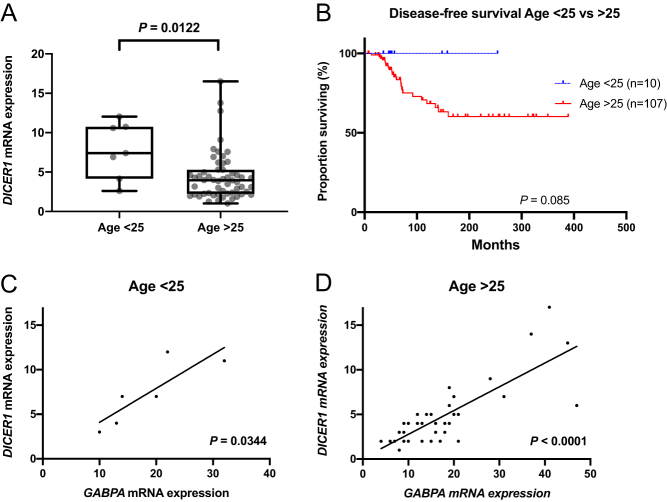
The age dependent differences in *DICER1* expression and survival. (A) *DICER1* mRNA expression in patients >25 years of age vs <25 years of age. (B) The disease-free survival in patients >25 years of age vs <25 years of age. (C) Correlation of *DICER1* and *GABPA* mRNA expression in patients <25 years of age. (D) Correlation of *DICER1* and *GABPA* mRNA expression in patients >25 years of age. A full colour version of this figure is available at https://doi.org/10.1530/ERC-19-0446.

The few *DICER1* mutations reported here occurred in young patients. Therefore, we evaluated the differences in *DICER1* mRNA expression between the groups. Higher *DICER1* mRNA expression was observed in patients younger than 25 years, an arbitrary cut-off selected by the authors (Fig. 8A). The disease-free survival in older patients seems as expected to be shorter (Fig. 8B). The correlation between *DICER1* mRNA expression and *GABPA* mRNA expression was also observed in both age groups (Fig. 8C and D).

## Discussion

In this study, we aimed to explore the effects of dysregulated *DICER1* in FTC and its interactions with the transcription factor *GABPA* based on recent findings in thyroid cancers (Yoo* et al.* 2016, Nicolson* et al.* 2018, Yuan* et al.* 2019). In our cohort of thyroid tumours, mutations in commonly affected regions of the *DICER1* gene seem to be a rare event. Interestingly, the few reported mutations in our cohort occurred in very young FTC patients, a 10- and 14-year-old female patient, respectively. Both showed missense mutations and *DICER1* mRNA expression in the top 25 percentile. Unfortunately, no constitutional DNA was available for testing of the 10-year-old patient, to determine the somatic or constitutional status of the mutation. The 14-year-old patient displayed two somatic mutations which were both present in heterozygous state as previously reported (Nicolson* et al.* 2018); however, no allelic separation was performed, and we cannot ascertain whether these mutations occurred in *cis* or in *trans*. Nevertheless, as both patients displayed missense mutations in the catalytic RNAse IIIb domain of *DICER1* in addition to the additional stop mutation found in one of the patients, the mutations are expected to be of impact as they might alter the function of the DICER1 protein. Overall, these results demonstrate that paediatric patients with thyroid cancer, in general, could mandate specific clinical consideration given their potential associations to genetic alterations not usually found in adult thyroid cancer.

We also searched the Cancer Genome Atlas (TCGA) database for *DICER1* mutations in follicular variant PTC (FV-PTC) patients from the cBio Cancer Genomics Portal. As FV-PTCs regularly display *RAS* family mutations and thereby bear some genetic similarities to follicular thyroid tumors, this was the most appropriate comparison as no *bona fide* FTC samples are displayed in the TCGA dataset. In the TCGA FV-PTC data set, one mutation in 107 cases has been reported, a missense p.R1906S mutation occurring in a 20-year-old female patient (TCGA-EM-A2CT). Thus, *DICER1* mutations seems to be rare, but possibly aggregating in young and FV-PTC FTC patients (2/11; 18% in our cohort). Intriguingly, *DICER1* mutations have just recently been reported in macrofollicular variants of FTCs as well as in poorly differentiated thyroid cancer specimen occurring in younger patients (Bongiovanni* et al.* 2020, Chernock* et al.* 2020).

We observed *DICER1* mRNA down-regulation in FTCs/HuCCs as well as in FTAs, and the results were confirmed at the protein level with weaker DICER1 immunoreactivity in FTCs compared to corresponding normal thyroid tissues. This observation was even more prominent in older patients and in HuCCs, suggesting that dysregulation of *DICER1* could be important in HuCC tumorigenesis and further endorse the separation of FTC and HuCC. Indeed, FTC and HuCC have recently been shown to dissent genetically (Ganly* et al.* 2013, Gopal* et al.* 2018, Corver & Morreau 2019). The *DICER1* down-regulation could also explain the observed worse prognosis in HuCC patients in our relatively small cohort, although it is in contrast with previous epidemiological findings that HuCC and FTC exhibit similar prognosis (Bhattacharyya 2003, Nagar* et al.* 2013). Moreover, the finding of down-regulated* DICER1* mRNA in FTAs compared to normal thyroid tissues suggests that aberrant regulation of this gene could be an early tumorigenic event. This assumption is indeed supported by the findings of multinodular goitre as the sole thyroid manifestation in subsets of patients with DICER1 syndrome, suggesting that *DICER1* gene aberrancies not always lead to overtly malignant phenotypes.

Previous studies have indicated that GABPA regulates *DICER1* through binding of the *DICER1* promoter (Yuan* et al.* 2019), thus acting as a regulator of this gene. In this study, we provide evidence of such an interaction in FTC. First, in our clinical material we observed a strong correlation between *GABPA* mRNA and *DICER1* mRNA expression. This is in line with observations in other types of thyroid cancer including FV-PTCs from the TCGA database (r = 0.5467; *P* value <0.001) and PTC in our previous report (Yuan *et al.* 2019). Secondly, after knocking down *GABPA*, we observed reduced *DICER1* expression on mRNA and protein levels in addition to a dysregulated mRNA machinery, and the former association was also visualized by overexpressing *GABPA* and observing an increase in DICER1 protein levels. Finally, through a ChIP assay we observed for the first time that GABPA indeed binds the *DICER1* promoter in FTC cell lines, which was expected given that previous results from our group displayed increased *DICER1* transcriptional activity upon GABPA overexpression using a luciferase reporter in anaplastic thyroid carcinoma cells (Yuan* et al.* 2019). Collectively, our data suggests that GABPA regulates the expression of *DICER1* and contributes to the downstream consequences of dysregulated *DICER1* (e.g. miRNA dysregulation, as observed in our cell lines). It should be stressed, however, that although the reduction in *GABPA* mRNA might explain the attenuated *DICER1* mRNA levels observed in our clinical samples, other independent mechanisms might silence the *DICER1* translational output. Interestingly, recent findings of oncogenic *miR-146b-5p* attenuating miRNA biosynthesis by targeting *DICER1* and reducing its expression has been implicated in thyroid cancer (Ramirez-Moya* et al.* 2019). As of this, there might be several synchronous mechanisms operational which all affect the total *DICER1* mRNA output.

Furthermore, our data suggest that GABPA regulates *DICER1*, in turn contributing to increased proliferation in FTC cell lines, as demonstrated by *DICER1* depletion and overexpression experiments. Intriguingly, in our FTC cell lines, overexpression of *DICER1* affected proliferation to a higher extent than *DICER1* depletion. The reasons for this discrepancy can only be speculated on, but since endogenous DICER1 protein expression in this cell line is low, augmentation of *DICER1* expression might give rise to a greater dose-dependent effect than what would be seen if depleting already low levels of *DICER1*. A recent study of *DICER1* in PTC by Ramírez-Moya and co-workers showed that down-regulation of *DICER1* promoted proliferation, migration, invasion and epithelial-mesenchymal transition through miRNA down-regulation and that *DICER1* indeed can act as a tumour suppressor (Ramirez-Moya* et al.* 2019). These data therefore support our findings of reduced proliferation upon *DICER1* overexpression in FTC cell lines and imply that *DICER1* could be a tumour suppressor gene of importance in follicular thyroid tumours.

The fact that DICER1 is a key enzyme in the miRNA maturation and that itself is regulated by miRNAs indicates exceptionally intricate mechanisms in DICER1 dysregulation and thyroid tumorigenesis. In FTC cell lines, we observed reduced expression levels of the thyroid-cancer associated miRNA *miR-34a-5p* upon *DICER1* or *GABPA* depletion, suggesting that components of the miRNA machinery are affected by the general *DICER1* down-regulation seen in FTCs. The *miR-34a-5p* has been shown to exhibit tumour-suppressive properties in PTC, although implicated, the role of *miR-34a-5p* is not fully understood (Yang* et al.* 2017). Of potential interest, *miR-34a-5p* targets *MDM4*, a p53 regulator that has been found down-regulated in thyroid cancer, with a coupling to advanced stages of the disease (Prodosmo* et al.* 2008). Moreover, the oncogene *MET*, which is frequently up-regulated in thyroid cancer, is also a predicted *miR-34a-5p* target (Ruco* et al.* 2001).

Clinically, we observed only small differences in survival in low or high *DICER1* expressing patients. However, we observed significant couplings between *DICER1* expression and patient age, with higher expression in younger patients. The *GABPA* and *DICER1* correlation was also observed in both age groups which indicates that GABPA regulates *DICER1* independently of age. Also, as observed before, *TERT* promoter mutations are more abundant in older patients (Liu* et al.* 2014). From a traditional molecular genetic perspective, the relationship between *GABPA* and *DICER1* is somewhat puzzling. Given the ambivalent actions of GABPA as a synchronous modulator of the tumour suppressor *DICER1* and the putative oncogene *TERT*, there might be dose-depending modes of action at play that we do not fully understand (Yuan* et al.* 2019). Indeed, the dualistic properties of GABPA in this context and whether it should be regarded as a tumor suppressor or oncogene in thyroid cancer will need to be addressed in future studies.

In summary, *DICER1* mutations in commonly mutated regions are rare in FTC but do occur in subsets of young patients. The down-regulation of *DICER1* expression, however, seems to be an early tumorigenic event and is at least, in part, regulated by the transcription factor GABPA. The *DICER1*-*GABPA* partnership constitutes a novel tumour suppressor axis in FTC with possible implications for future therapeutic interventions using miRNA-based strategies.

## Supplementary Material

Supplementary Figure 1. (A) A schematic illustration of DICER1 domains with three mutations identified in two FTC patients. The blue and green lines refer to two separate patients. (B) Results from the DICER1 hotspot mutational screening in follicular thyroid tumours.

Supplementary Figure 2. GABPA over-expression increases DICER1 protein expression in FTC-238 cells. The DICER1/GAPDH quote denotes the ECL signal intensity quote between these proteins relative to vector control. Size ladder is indicated on the right of the blots.

Supplementary Figure 3. Evaluation of the effect of DICER1 depletion on cell growth in the FTC 238 cell line using trypan blue exclusion assay. An increase in cell number was observed after DICER1 depletion at three different time points.

Supplementary Figure 4. Evaluation of the effect of GABPA depletion on apoptosis in FTC-238 cells. Representative flow cytometric images of FTC-238 cells stained with annexin V and propidium iodide (PI). Early apoptosis is represented by annexin V+/PI- (lower right quadrant), and late apoptosis is indicated by annexin V+/PI+ (upper right quadrant).

Supplementary Table 1. Primers and siRNA sequences

Supplementary Table 2. Top 20 miR-34a-5p targets predicted by the mIRDB database

## Declaration of interest

The authors declare that there is no conflict of interest that could be perceived as prejudicing the impartiality of the research reported.

## Funding

This work was supported by the Swedish Cancer Society, the Swedish Society for Medical Research, the Cancer Research Funds of Radiumhemmet, Karolinska Institutet and the Stockholm County Council.
