# Supplementary material for: GABPA-dependent down-regulation of DICER1 in follicular thyroid tumours
Source: Endocr Relat Cancer. 2020 Mar 11;27(5):295–308. doi: 10.1530/ERC-19-0446 (PMC7159166; doi:10.1530/ERC-19-0446)
Supplement: Supplementary Figure 1. (A) A schematic illustration of DICER1 domains with three mutations identified in two FTC patients. The blue and green lines refer to two separate patients. (B) Results from the DICER1 hotspot mutational screening in follicular thyroid tumours. [file supplementary_figure_1.pdf]

A

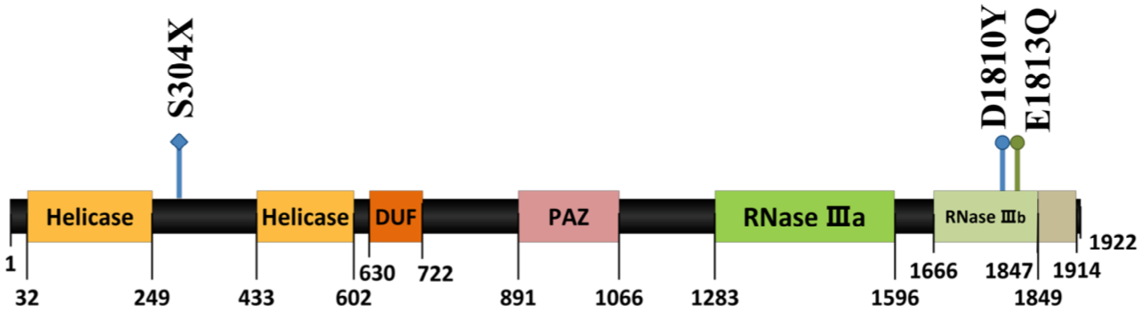

B

	Codon						
	**290**	**304**	**1705**	**1709**	**1810**	**1813**	**1814**
**Tumour type**							
FTC	0/61	**1/61**	0/53	0/53	**1/61**	**1/61**	0/61
HuCC	0/15	0/15	0/13	0/13	0/15	0/15	0/15
FTA	0/43	0/43	0/43	0/43	0/43	0/43	0/43
FT-UMP	0/7	0/7	0/7	0/7	0/7	0/7	0/7

FTC, follicular thyroid carcinoma
HuCC, Hürthle cell carcinoma
FTA, follicular thyroid adenoma
FT-UMP, follicular tumour of uncertain malignant potential

Supplementary Figure 1. (A) A schematic illustration of *DICER1* domains with three mutations identified in two FTC patients. The blue and green lines refer to two separate patients. (B) Results from the *DICER1* hotspot mutational screening in follicular thyroid tumours.
