# Supplementary material for: GABPA-dependent down-regulation of DICER1 in follicular thyroid tumours
Source: Endocr Relat Cancer. 2020 Mar 11;27(5):295–308. doi: 10.1530/ERC-19-0446 (PMC7159166; doi:10.1530/ERC-19-0446)
Supplement: Supplementary Figure 2. GABPA over-expression increases DICER1 protein expression in FTC-238 cells. The DICER1/GAPDH quote denotes the ECL signal intensity quote between these proteins relative to vector control. Size ladder is indicated on the right of the blots. [file supplementary_figure_2.pdf]

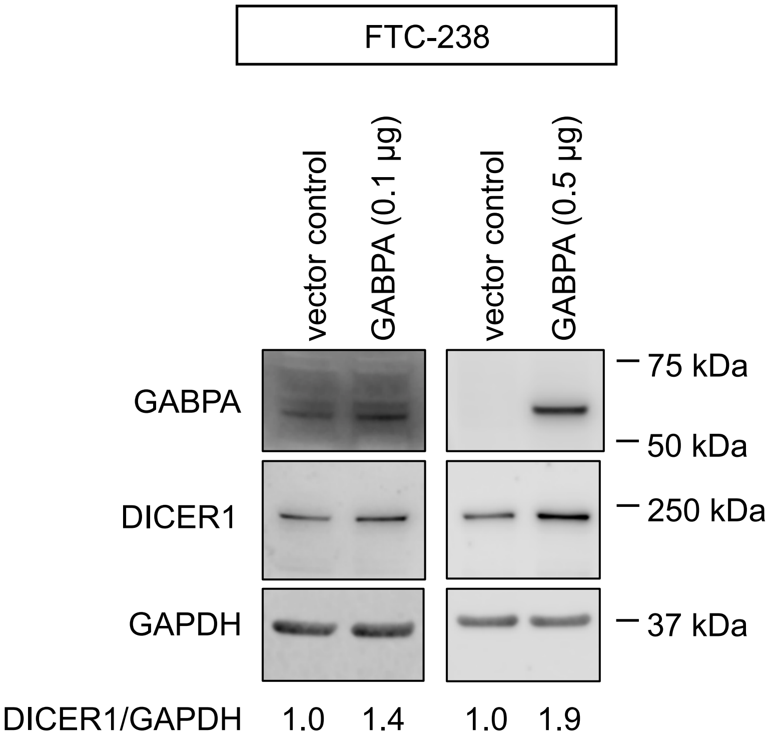

Supplementary Figure 2. GABPA over-expression increases DICER1 protein expression in FTC-238 cells. The DICER1/GAPDH quote denotes the ECL signal intensity quote between these proteins relative to vector control. Size ladder is indicated on the right of the blots.
