# Supplementary material for: GABPA-dependent down-regulation of DICER1 in follicular thyroid tumours
Source: Endocr Relat Cancer. 2020 Mar 11;27(5):295–308. doi: 10.1530/ERC-19-0446 (PMC7159166; doi:10.1530/ERC-19-0446)
Supplement: Supplementary Figure 3. Evaluation of the effect of DICER1 depletion on cell growth in the FTC 238 cell line using trypan blue exclusion assay. An increase in cell number was observed after DICER1 depletion at three different time points. [file supplementary_figure_3.pdf]

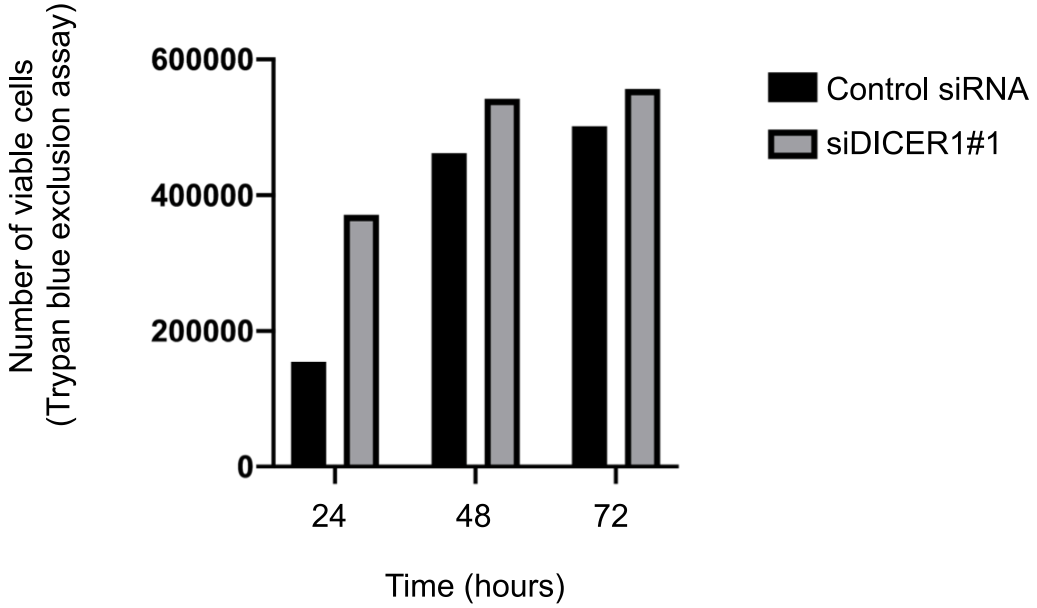

Supplementary Figure 3. Evaluation of the effect of *DICER1* depletion on cell growth in the FTC 238 cell line using trypan blue exclusion assay. An increase in cell number was observed after *DICER1* depletion at three different time points.
