# Supplementary material for: GABPA-dependent down-regulation of DICER1 in follicular thyroid tumours
Source: Endocr Relat Cancer. 2020 Mar 11;27(5):295–308. doi: 10.1530/ERC-19-0446 (PMC7159166; doi:10.1530/ERC-19-0446)
Supplement: Supplementary Figure 4. Evaluation of the effect of GABPA depletion on apoptosis in FTC-238 cells. Representative flow cytometric images of FTC-238 cells stained with annexin V and propidium iodide (PI). Early apoptosis is represented by annexin V+/PI- (lower right quadrant), and late apoptosis is i [file supplementary_figure_4.pdf]

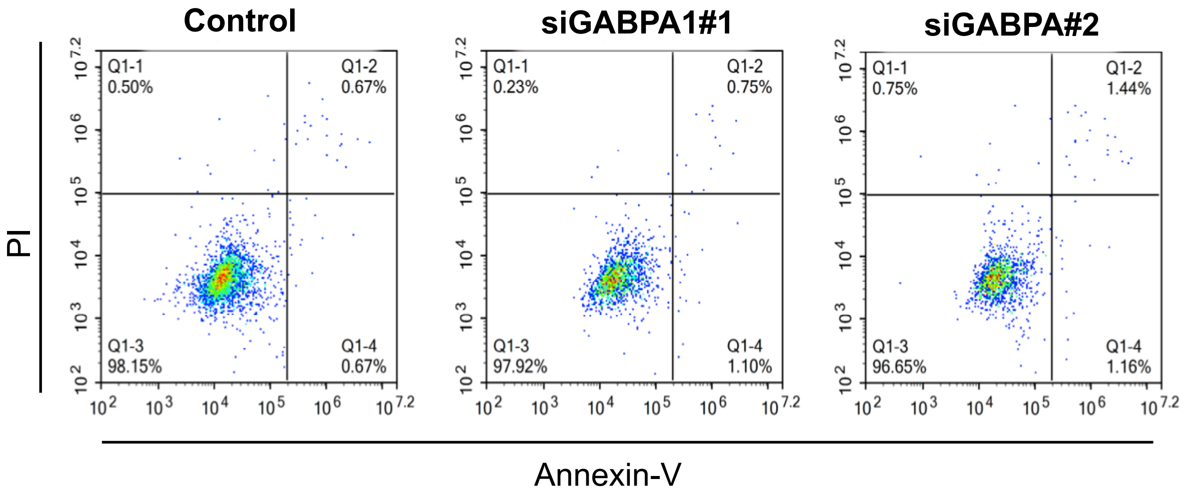

Supplementary Figure 4. Evaluation of the effect of GABPA depletion on apoptosis in FTC-238 cells. Representative flow cytometric images of FTC-238 cells stained with annexin V and propidium iodide (PI). Early apoptosis is represented by annexin V^+/PI^- (lower right quadrant), and late apoptosis is indicated by annexin V^+/PI^+ (upper right quadrant).
