# Supplementary material for: GABPA-dependent down-regulation of DICER1 in follicular thyroid tumours
Source: Endocr Relat Cancer. 2020 Mar 11;27(5):295–308. doi: 10.1530/ERC-19-0446 (PMC7159166; doi:10.1530/ERC-19-0446)
Supplement: Supplementary Table 1. Primers and siRNA sequences [file supplementary_table_1.pdf]

Supplementary Table 1. Primers and siRNA sequences

**siRNA**	
siGABPA#1	5'-GGAGCUGAUAGAAAUUGAGAUUGAU-3'
siGABPA#2	5'-GCAGAGUGCACAGAAGAAAGCAUUG-3'
siDICER1#1	5'-GAGUAAUGCUGAAACUGCAACUGAC-3'
siDICER1#2	N/A
**mRNA expression**	
GABPA primer F	5’- AAGAACGCCTTGGGATACCCT-3’
GABPA primer R	5’- GTGAGGTCTATATCGGTCATGCT-3’
DICER1 primer F	5’-CCTAGACCACCCCTATCGAGA-3’
DICER1 primer R	5’-CAGGTCAGTTGCAGTTTCAGCA-3’
β-actin primer F	5'-GCGGGAAATCGTGCGTGACAT -3'
β-actin primer R	5'-TGGCGTACAGGTCTTTGCGGATG-3'
**Sanger sequencing**	
Codon 290 primer F	5'-AAGACCTTAACTAAAATGCAAAGAAAAGAGC-3'
Codon 290 primer R	5'-TGGTGGATTGTGGACCATTTACTG-3'
Codon 304 primer F	5'-ACACCAGGGTCCCAGAACTA-3'
Codon 304 primer R	5'-GAGCCATTTGAGAACAACTGAAATCC-3'
Codon 1705&1709 primer F	5'-CTTGTGCACAAGCTTACGGTTCCA-3'
Codon 1705&1709 primer R	5'-CAGCGATGCAAAGATGGTGTTGT-3'
Codon 1810-1814 primer F	5'-TCTTTGTGAACTTTTCCCCTTTGA-3'
Codon 1810-1814 primer R	5'-TGGTATTTCCCCCCTTACTAAAGAA-3'
**ChIP - PCR and sequencing**	
DICER1 promoter primer F	5'-GGGCCACCATCTATTTCTCAT-3'
DICER1 promoter primer R	5'-GCCTGCGTTTCCTCGCGTT-3'
