# Supplementary material for: GABPA-dependent down-regulation of DICER1 in follicular thyroid tumours
Source: Endocr Relat Cancer. 2020 Mar 11;27(5):295–308. doi: 10.1530/ERC-19-0446 (PMC7159166; doi:10.1530/ERC-19-0446)
Supplement: Supplementary Table 2. Top 20 miR-34a-5p targets predicted by the mIRDB database [file supplementary_table_2.pdf]

Supplementary Table 2. Top 20 *miR-34a-5p* targets predicted by the mIRDB database

Target Rank	Target Score	miRNA Name	Gene Symbol	Gene Description
1	100	*miR-34a-5p*	*MDM4*	MDM4, p53 regulator
2	100	*miR-34a-5p*	*DLL1*	delta like canonical Notch ligand 1
3	100	*miR-34a-5p*	*RAP1GDS1*	Rap1 GTPase-GDP dissociation stimulator 1
4	100	*miR-34a-5p*	*FAM167A*	family with sequence similarity 167 member A
5	100	*miR-34a-5p*	*HCN3*	hyperpolarization activated cyclic nucleotide gated potassium channel 3
6	100	*miR-34a-5p*	*SDK2*	sidekick cell adhesion molecule 2
7	100	*miR-34a-5p*	*FAM76A*	family with sequence similarity 76 member A
8	99	*miR-34a-5p*	*E2F5*	E2F transcription factor 5
9	99	*miR-34a-5p*	*FKBP1B*	FKBP prolyl isomerase 1B
10	99	*miR-34a-5p*	*PPP1R11*	protein phosphatase 1 regulatory inhibitor subunit 11
11	99	*miR-34a-5p*	*SCN2B*	sodium voltage-gated channel beta subunit 2
12	99	*miR-34a-5p*	*MGAT4A*	alpha-1,3-mannosyl-glycoprotein 4-beta-N-acetylglucosaminyltransferase A
13	99	*miR-34a-5p*	*NAV3*	neuron navigator 3
14	99	*miR-34a-5p*	*SYT1*	synaptotagmin 1
15	98	*miR-34a-5p*	*SATB2*	SATB homeobox 2
16	98	*miR-34a-5p*	*CELF3*	CUGBP Elav-like family member 3
17	98	*miR-34a-5p*	*XYLT1*	xylosyltransferase 1
18	98	*miR-34a-5p*	*FLOT2*	flotillin 2
19	98	*miR-34a-5p*	*MET*	MET proto-oncogene, receptor tyrosine kinase
20	98	*miR-34a-5p*	*MYCN*	MYCN proto-oncogene, bHLH transcription factor
